# Humanoid Intelligent Display Platform for Audiovisual Interaction and Sound Identification

**DOI:** 10.1007/s40820-023-01199-y

**Published:** 2023-10-09

**Authors:** Yang Wang, Wenli Gao, Shuo Yang, Qiaolin Chen, Chao Ye, Hao Wang, Qiang Zhang, Jing Ren, Zhijun Ning, Xin Chen, Zhengzhong Shao, Jian Li, Yifan Liu, Shengjie Ling

**Affiliations:** 1 School of Physical Science and Technology, ShanghaiTech University, 393 Middle Huaxia Road, Shanghai, 201210 People’s Republic of China; 2 State Key Laboratory of Molecular Engineering of Polymers, Department of Macromolecular Science, Laboratory of Advanced Materials, Fudan University, Shanghai, 200433 People’s Republic of China; 3 School of Textile and Clothing, Yancheng Institute of Technology, Jiangsu, 224051 People’s Republic of China; 4 School of Textile Science and Engineering, Wuhan Textile University, Wuhan, 430200 People’s Republic of China; 5 Shanghai Clinical Research and Trial Center, 201210 Shanghai, People’s Republic of China

**Keywords:** Silk fibroin, Ionoelastomer, Luminescent display, Machine learning, Audiovisual interaction

## Abstract

This study proposes a rational strategy for the design, fabrication and system integration of the humanoid intelligent display platform (HIDP) to meet the requirements of highly humanized mechanical properties and intelligence for human–machine interfaces. The platform's sandwich structure comprises a middle light-emitting layer and surface electrodes, which consists of silicon elastomer embedded with phosphor and silk fibroin ionoelastomer, respectively. Both materials are highly stretchable and resilient, endowing the HIDP with skin-like mechanical properties and applicability in various extreme environments and complex mechanical stimulations. Furthermore, by establishing the numerical correlation between the amplitude change of animal sounds and the brightness variation, the HIDP realizes audiovisual interaction and successful identification of animal species with the aid of Internet of Things (IoT) and machine learning techniques. The accuracy of species identification reaches about 100% for 200 rounds of random testing. Additionally, the HIDP can recognize animal species and their corresponding frequencies by analyzing sound characteristics, displaying real-time results with an accuracy of approximately 99% and 93%, respectively. In sum, this study offers a rational route to designing intelligent display devices for audiovisual interaction, which can expedite the application of smart display devices in human–machine interaction, soft robotics, wearable sound-vision system and medical devices for hearing-impaired patients.

**Supplementary Information:**

The online version contains supplementary material available at 10.1007/s40820-023-01199-y.

## Highlights

- A humanoid intelligent display platform (HIDP) is created using stretchable and resilient ionotronic materials, and can be applicable in extreme environments and complex mechanical stimulations.
- HIDP links sound amplitude and brightness through machine learning for audiovisual interaction.
- HIDP identifies and displays animal species and corresponding frequencies in real-time.

**Supplementary Information:**

The online version contains supplementary material available at 10.1007/s40820-023-01199-y.

## Graphical abstract

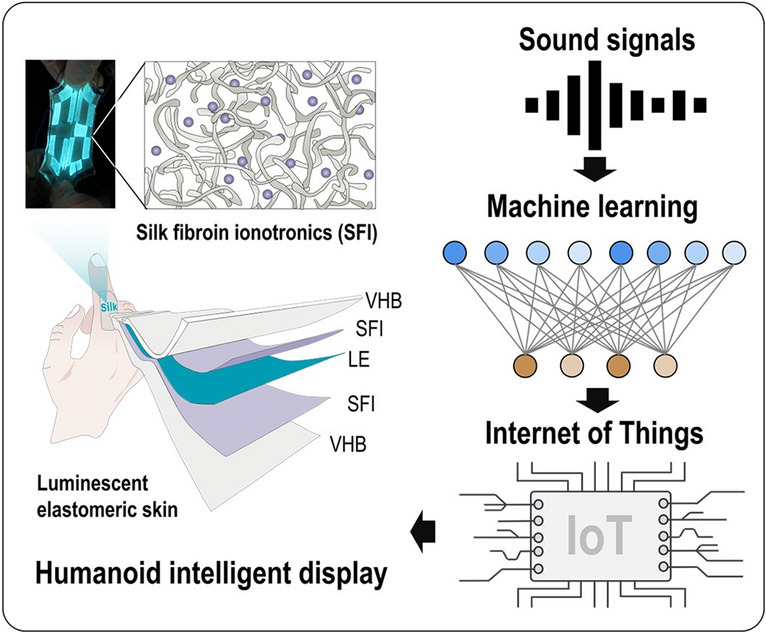

## Introduction

Display platforms in the human–machine interfaces need to be highly humanized, especially in terms of mechanics and functionality [1–3]. Mechanically, they should have the same tactile and mechanical properties as the human skin, i.e., soft physical form and good elasticity, capable of conformal fit with surfaces of different topologies [4]. In daily use, the platforms should withstand complex mechanical and environmental stimuli, such as stretching, twisting and bending. These mechanical stimuli may be long-term or transient and single-time or long-term cyclic. After these complex mechanical stimuli are removed, the light-emitting device in the display needs to quickly return to its original shape to maintain the display's accuracy. In some extreme applications, such as visualized injury warning [5] and outdoor robots [6], these light-emitting devices are even required to be robust against extreme damage such as corrosion, needle-punching, and cutting, and should have self-healing ability after being partially damaged. In terms of their function, light-emitting devices need to eliminate the constraints of the passive display, and are expected to be integrated with artificial intelligence and IoT technologies to realize intelligent interaction between the display platform and the environment, human body, and machines [7–13]. For example, display devices should have human-like audiovisual ability to analyze and process audiovisual information. Such capabilities can enable the realization of human–computer interactive display, question-and-answer display, thinking display, and even real-time dialogue display for semantic communication.

However, the existing display technologies are still far from meeting these human-like requirements. For example, the structural and mechanical properties of the existing display materials are significantly different from those of the skin [4]. Therefore, only limited materials and technologies with significant shortcomings can be used to achieve even the stretchability of display devices. For example, stretchable, bendable and foldable display platforms are constructed usually by assembling light-emitting diodes on an elastic substrate to form a serpentine or buckle meta-structure [14–17]. However, as the LED is not stretchable, its effective use-stretch ratio is still less than 120% that is considerably lower than the stretchability of skin or elastomers [1, 18]. In addition, electroluminescent (EL) devices obtained by combining phosphor particles with elastomers have also been proven to be a practical approach for preparing highly stretchable display devices [19, 20]. However, in most of the reported design strategies, the stretchability of the devices is determined by the electrode layer rather than the light-emitting layer. The former layer in these systems is usually a composite made of elastomeric polymers and conductive nanomaterials, such as silver nanowires [19, 21], carbon nanotubes [22, 23], and graphene [24]. Therefore, the connectivity of these nanomaterials will be lower than the percolation threshold once the tensile strain exceeds a certain value, which causes a sharp decline in conductivity and affects the device's luminescent performance [25, 26]. Consequently, the effective use-stretch ratio of such devices is usually in the range of 20%-150% [19, 21–24, 27–29]. In addition, such nanostructures cannot restore the original connectivity in case of damage, therefore, these devices often suffer from functional instability or even failure during cyclic stretching [30].

In terms of functionality, current intelligent display techniques are also unable to meet the demand for human–machine interfaces. For instance, current smart display devices are mainly based on tactile perception and response [8, 31–33]. The tactile perception can be achieved through capacitive [5, 34], piezoelectric [31] and triboelectric responses [35, 36]. Therefore, these devices are primarily used for tactile sensing [7], motion monitoring [33, 37, 38], injury warning [5], safety surveillance [35], etc. However, tactile perception requires a direct contact between objects and display devices, and consequently the types of objects and environments that can be sensed are relatively limited. In addition, these devices cannot communicate and interact easily with the environment, human body and machines. These issues can be overcome by using optical and acoustic signals to realize the interaction between the display device and the environment. However, the related research has not been reported yet in published literature.

Therefore, this paper proposes to develop a humanoid intelligent display platform (HIDP), which has mechanical properties like human skin. Furthermore, it should be able to withstand harsh scenarios, such as low temperature, high temperature, and water, as well as complex mechanical stimulus, such as stretch, squeeze, crimp and long-term cyclic loading. In addition, the platform should have human-like audiovisual recognition and interaction abilities. For instance, it is necessary to realize semantic-distinguishable, question-and-answer, and conversational display through audiovisual interaction. The implementation of HIDP makes it possible to accelerate intelligent display device applications in human–machine interface, soft robotics, wearable sound-vision system and medical devices for hearing-impaired patients.

## Experimental

### Preparation of Silk Fibroin Ionoelastomer (SFIE) Electrode

The preparation of SFIE electrode mainly includes three parts. First, the degumming process is carried out, specifically, *Bombyx mori* silkworm cocoons (20 g) are boiled in 0.5% (w/w) NaHCO_3_ solution (4 L) for 30 min, and this process is repeated to thoroughly remove the sericin. Subsequently, the degummed silk fibers are washed with distilled water and dried in an oven at 60 °C for 12 h. Second, LiCl (2, 2.5, 3.3, 4 and 5 g) are dissolved in formic acid (FA, 98%, 100 g), respectively, followed by the addition of degummed silk fibers (10 g) and stirred vigorously at room temperature for 1 h, and then filtered by gauze to remove impurities. The related mass ratio of SF/LiCl in silk fibroin ionotronics (SFI) is varied from 5:1 to 2:1, respectively. Last, the SFI electrode is molded, and the above dissolved solution is poured into a 30 × 20 cm^2^ silicone rubber mold after stationary placement for 0.5 h. The FA solvent is evaporated in a chemical fume hood at a relative humidity (RH) of about 80%. The RH is kept at about 80% with a high power humidifier for three days, which incubates SFI electrode to fabricate a resulting SFIE. After completing the above operations, the SFIE is stored in a drying box. For the characterization experiments except for luminance test, the mass ratio of SF/LiCl is set at 5:2.

### Fabrication of HIDPs

The electroluminescent (EL) solution is prepared by mixing EL phosphor powder (ZnS, Shanghai Keyan Phosphor Technology Co., Ltd) with Ecoflex 00–30 (Smooth-On, Inc) in a ratio of 1:1. The as-prepared solution is stirred thoroughly for 15 min, subsequently spin-coated into a film on polystyrene substrates at rates of 1000, 1500, 2000 and 3000 rpm, respectively, followed by curing at room temperature for 1 h. And the resulting thickness of EL film is 105, 83, 58 and 40 μm, respectively. Subsequently, both surfaces of the EL layer are treated with plasma cleaning at 10 W for 40 s to strengthen the interface bonding between the SFIE and EL layer. Eventually, the HIDP with a sandwich structure is prepared by assembling the EL layer and two SFIE electrodes. The patterned HIDPs are prepared by punching the SFIE electrode into various patterns via printing molds and assembling with the EL layer.

### SFIE Characterization

The optical transmittance of SFIE (SF: LiCl = 5:2, thickness: 0.1 mm) is measured using a UV–Visible-NIR spectrophotometer (Agilent Cary 5000) with a scanning range of 380–800 nm. The mechanical testing of SFIE samples is performed by a mechanical testing machine (Instron 5966 machine, Instron, Norwood, USA) with a tensile rate equal to 50 mm min^−1^. Both sides of samples are installed on the testing machine. The height of the frame is adjusted to keep the samples at zero load point, and the initial length of the samples is measured with a caliper. The peel test of the SFIE sample is also carried out by the mechanical testing machine. The SFIE electrode with/without the PS substrate is tightly fitted to the pig skin, respectively. Both sides of samples are installed on the testing machine testing machine for the peel test, and the tensile rate is 10 mm min^−1^. Tensile measurements are carried out at 20 °C and 44%RH.

### HIDP Characterization

The cross-sectional morphology of HIDP device is observed by a high-resolution scanning electron microscope (SEM, JEOL JSM-7800F, Tokyo, Japan) at an acceleration voltage of 1 kV. Samples are coated with a 5 nm thick gold layer to provide conductivity before observation. The capacitance of the electrical double layer and light-emitting layer is measured with an LCR digital bridge (TH2830) between frequencies ranging from 50 Hz to 100 kHz. The luminance of the HIDP device (the mass ratio of SF/LiCl is from 5:1 to 2:1) is measured by using a PR-655 photometer (Photoresearch), and the related alternating voltage signals are generated using a function generator (FY8300S-60 M) and a high-voltage amplifier (LPA400B). All the luminous tests are carried out in a dark environment.

Mechanical testing of the HIDP samples is performed using a mechanical testing machine (Instron 5966 machine, Instron, Norwood, USA). To apply an alternating electric field during the stretching process, both ends of the fixture are pasted with copper sheets as the external connection electrode of the HIDP to apply alternating voltage signals. Both sides of samples are installed on the copper sheets. The height of the frame is adjusted to keep the samples at the zero load point, and the initial length of the samples is measured with a caliper. The luminescence change of the HIDP devices is observed synchronously during the stretching process with a tensile rate of 50 mm min^−1^ using a camera (Canon, EOS 80D). The experimental steps of observing the luminous state during cyclic stretching test are the same as described above, where the tensile rate, strain and cycle times are set at 200 mm min^−1^, 200% and 100 cycles, respectively.

Following the aforementioned experimental method, the luminescence changes of HIDP devices with pre-notches (1 mm) are recorded in situ using a camera (Canon, EOS 80D) during the tensile test. In this test, a tensile rate of 50 mm min^−1^ is used, and the cyclic stretching test is carried out with tensile rate, strain and cycle times set at 200 mm min^−1^, 200% and 100 cycles, respectively.

### Machine Learning Classification of Brightness Changes for Animal Voice Identification

The raw data of animal voices were 5 segments of audio, 2 min for each animal. The audio was perceived by the sound sensor when playing, and the sound intensity (400–1023) was converted into an electrical signal through Arduino board, then the voltage (0–5 V) signal was divided into 4 levels and triggered the flashing of HIDP with a voltage amplifier. The flashing was recorded as a video with a frame rate of 30 and a length of 2 min. In machine learning research for classification from flash recording, we selected interest points on HIDP randomly and collected the RGB values changes of these points within 5 s. Therefore, the shape of one piece of data was (150, 3). Each video was sampled 200 times to generate a dataset containing 1000 pieces of data.

The compilation of machine learning algorithm was carried out with TensorFlow (2.4.0). The neural network model took the RGB values changes data as input and outputted the binary codes of correspond animals. The training process was set with cross entropy loss function and Adam optimizer. After 500 epochs, the performance of the model on both the training set (350) and validation set (150) was similar, with an accuracy of nearly 100%.

In actual testing, we used functions from OpenCV (4.6.0) to capture real-time images captured by the camera. We set the mouse click event to activate the sampling program at any time and location, captured the 5-s RGB values changes of several points of interest. The program took data from all collection points into the model prediction, and give the average probability of belonging to each animal.

### Code Compilation for Deep Learning and Data Analysis of Animal Sounds

The models for recognizing the species and corresponding frequencies of animal vocalizations are compiled using Python (3.10.8) and TensorFlow (2.11.0). A total of 4586 pieces of audio from five types of animal sounds (bird, cat, dog, elephant and tiger) with different frequencies are used for training the classification models for predicting the species and frequencies of animal sounds. For testing, 242 sets of animal sounds are used. A sound (barking, meowing, etc.) is characterized as a vibration that propagates through a medium, which can be converted to an electrical signal through the use of a transducer, such as a microphone. This signal can then undergo further preprocessing and analysis using machine learning techniques. Sound waves are rich in information that includes frequency, amplitude and direction. Features from this information can be extracted and utilized for learning. Therefore, the one-dimensional signal is converted to Mel-spectrogram [39] for deep learning. The Mel-spectrogram is an image representation of the audio signal that converts frequency into a Mel scale, which can be used as the input to our machine learning model. The Mel-spectrogram can be used for providing the sound information similar to what a human would perceive. The Mel scale is related to the frequency in Hertz by the following equation:

1 $$m = 2595\log_{10} \left( {1 + \frac{f}{700}} \right)$$

where *m* represents the Mel-frequency and *f* represents the real frequency.

To standardize the signal, zero-padding is implemented prior to feature extraction. The sampling rate is all set to 16,000, whereas the duration remains constant at 10.02 s. The audio library called Librosa is applied to extract the Mel-spectrogram from the raw audio waveforms. Following this process, each input sample has a shape of 128 × 627. The machine learning models are expected to learn features from the images of the Mel-spectrogram. The machine learning model is based on Res-Nets-50v2, which is a 50-layer CNN. It contains 1 MaxPool layer, 48 convolutional layers, and 1 average pool layer. The Adam optimizer is used with a learning rate of 0.001. A total of 200 batches are trained to test and save the model, including prediction model and network weight. To adapt to the input with other audio lengths, the input shape of models is set to (128, None, 1).

### Customized Tkinter Interface

A graphical user interface (GUI) is built using tkinter in Python (3.10.8), deploying the classifications models as aforementioned. Specifically, the GUI receives a sound signal from a transducer (a microphone in this study) and converts it into a Mel-spectrogram. The CNN model receives the Mel-spectrogram for prediction and encodes the result to ‘utf-8’. The HIDP then simultaneously receives and displays the prediction results. In addition, the tkinter GUI synchronously displays the prediction results. At the HIDP interface, “b” refers to bird, “C” refers to cat, “d” refers to dog, “E” refers to elephant, and “t” refers to tiger. The numbers 1 ~ 9 refers to the frequency of animal sounds, and 0 refers to invalid audio.

## Results and Discussion

### Design of HIDP

**Fig. 1:**
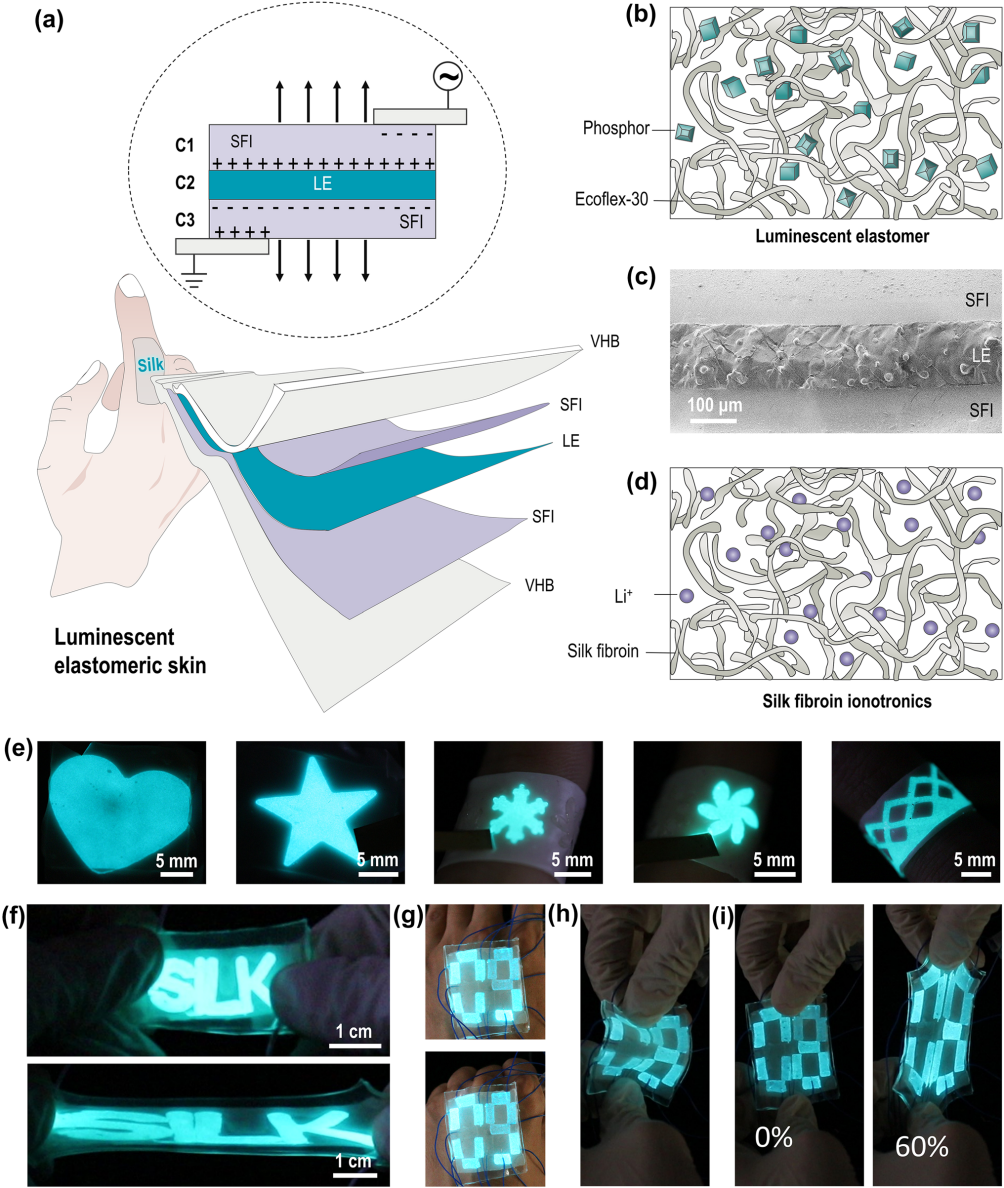
Design and images of HIDP. **a** Schematic of the structure and working principle of the HIDP. **b** Schematic of the luminescent elastomer structure. **c** Cross-sectional SEM image of the HIDP. **d** Schematic of the silk fibroin ionotronics structure. **e** Photographs of HIDPs with various patterns. **f** Photographs of “silk”-patterned HIDP before (top) and after (bottom) being stretched to three times its original length. **g** Photographs illustrating the digital-patterned HIDP (4 cm × 5 cm) attached to the back of the hand are deformed by stretching (top) and bending (bottom) of the palm. **h** Photograph of the digital-patterned HIDP under bending. **i** Photographs of the digital-patterned HIDP before (left) and after (right) being stretched under the strain of 60%

The light-emitting device designed here adopts a sandwich structure as shown in Fig. 1a. The middle layer is the light-emitting layer, and the two electrode layers cover the top and bottom surfaces as illustrated in Figs. 1b-d. Such structural design can not only simplify the device but also contribute to the realization of skin-like mechanical properties and tactile sensation. This is because all three layers can be made of highly stretchable materials. The simplified sandwich structure also makes it easier to prepare larger display screens compared to other more complex designs. More specifically, a silicone/ZnS composite membrane produced by spinning coating is adopted for the luminous layer, as shown in Figs. 1b and S1. In this composite, silicone is the structural support with mechanical properties similar to the human skin. For example, the strength and modulus of both silicon and human skin are within the range of 0.2–0.5 and 0.1–0.7 MPa, and both are resilient under stretching. The embedded ZnS particles form the luminous component, which can be activated by an alternating current (AC) electric field. In addition, the spin coating is adopted because it can reasonably fix the thickness of the membrane by controlling the amount of dispersion used. This is especially important for the preparation of ultra-thin membranes.

The electrode layers are made of silk fibroin ionotronics (SFI), a material that consists of silk fibroin, Li^+^ ions, and water. Compared with the electron-type stretchable elastomers, the SFI has many advantages as the electrode layer of HIDP [40–43]. First, SFI has a material composition similar to skin: both are based on the structural framework of proteins and contain a certain amount of water and ions. The ions and waters in both SFI and skin systems can act as charge carriers to realize the ionic and hydration proton conduction [43]. Second, SFI has excellent optical transparency and its transmittance is higher than 85% in visible wavelengths as shown in Fig. S2. This high transmittance ensures high fidelity of the display. Third, the components of the SFI are biodegradable and biocompatible, therefore, the application of its devices can be extended to artificial skin, and even to biomedicine and other related fields. Last, thanks to the unique ionic and hydrating proton conduction mechanism independent of the percolation threshold [43], the SFI, similar to other ionotronics, can maintain its conductivity even under ultrahigh tensile strain. This behavior is in sharp contrast to the failure of electronic stretchable electrodes at high stretch ratios. In addition, the inherent advantages of ionotronics, such as frost resistance, adhesion, notch insensitivity, and self-healing, provide great benefits to the assembly of HIDPs and the application of these devices in harsh environmental conditions [13, 44–46].

However, SFIs are viscoelastic rather than elastic. The viscoelasticity is beneficial for its assembly with a luminescent layer, as it ensures a tight fit between SFI and luminescent layer after the hydrophilic treatment of the luminescent layer, as shown in Fig. S1. Indeed, as Fig. S3 shows, the spline bonded by the two materials can withstand a weight of 20 g, which is about 200 times its own mass, and the bonded interface remains stable without separating or tearing. In contrast, viscoelastic materials are readily deformed under small external forces and cannot recover their initial shape after the removal of the external force. Therefore, the hygroscopic-induced crystallization strategy is introduced here to regulate the crosslinking state of the silk molecular network [40]. As for silk fibroin, the crystallization, i.e., conformational transition, forms β-sheet nanocrystals, which refer to a structure that serves as the crosslinker of the molecular network to limit the slippage of amorphous chains. Therefore, the transition of SFI from viscoelasticity to the elasticity can be achieved by controlling the degree of crystallinity of SF, thereby preparing silk fibroin ionoelastomer (SFIE) with mechanical properties and elasticity that match those of the human skin.

Detailed experimental procedures for conducting hygroscopic induced crystallization strategy are described in the experimental section. In short, this strategy incubates the SFI in a sealed high-humidity environment (relative humidity ≈ 80%) for three days. During this process, the strong hygroscopicity of lithium ions drives them to absorb a large amount of water from the surroundings and thereby causes the amorphous SF network to swell. The resulting SFIE can thus be regarded as a highly concentrated polymer solution in which the free water can be easily transported over the whole amorphous network. In this case, a significant plasticization of free water reduces the glass transition temperature of SF to a temperature considerably below the room temperature. Hence, the conformational transition of SF from the random coil into β-sheet nanocrystals can occur at room temperature. The crystallization of SF terminates when the crystal crosslinking density reaches a certain level, where the free volume between SF molecules cannot accommodate the motion of their chain segments, as shown in Fig. 1d. The resulting SF network thus features a dual network structure with dense entanglements and sparse nanocrystals. The former enables the transmission of tension in a polymer chain along its length and to many other chains [47]. Meanwhile, the latter prevent the polymer chains from disentangling. Accordingly, the resulting SFIE has a high stretchability and excellent elasticity. After being stretched to three times the original length, it can recover to 87.5% within 10 s, and fully recover after 70 s. This is shown in Fig. S4a.

### Mechanical Performance of HIDP

**Fig. 2:**
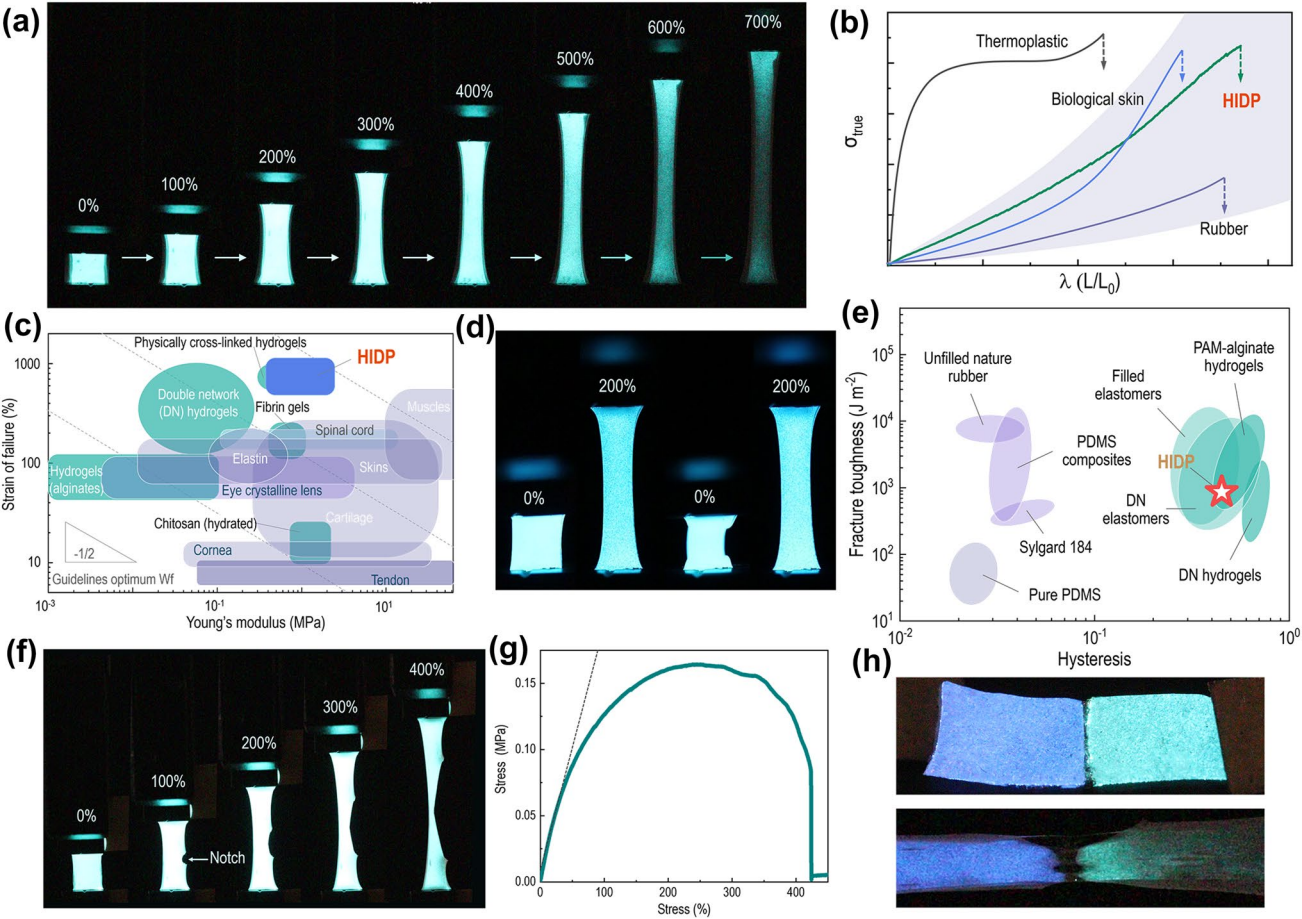
Mechanical performance of the HIDP. **a** Snapshots of the HIDP under stretching. **b** True stress-elongation curves of the HIDP, thermoplastic materials, biological skin, and rubber. The true stress-elongation curves of thermoplastic materials, biological skin, and rubber are adapted with permission [73]. Copyright 2018, The American Association for the Advancement of Science. **c** Comparison of Young’s modulus and strain of failure of the HIDP, and other representative ionotronics material. The Ashby plot of representative ionotronics materials has been adapted with permission [74]. Copyright 2014, Royal Society of Chemistry. **d** Photographs of the HIDP at the first (first two) and 100th stretching cycle (last two) with a stretch amplitude of 200%. **e** Comparison of the hysteresis and fracture toughness of the HIDP and other elastomers. The Ashby plot of other elastomers has been adapted with permission [75]. Copyright 2019, Proceedings of the National Academy of Sciences. **f** Snapshots of the pre-notched HIDP during the stretching process. **g** Tensile stress–strain curve of the pre-notched HIDP. **h** Photographs of the dual-color HIDP at the strain of 0% and 300%

When SFIE and light-emitting layer are integrated, their skin-like mechanical properties are also retained in the resulting HIDP, which has a skin-like tactile impression, stretchability and elasticity. In addition, it can be mechanically punched or cut into desired shapes as shown in Figs. 1e-i, or assembled into a stretchable luminescent display device through compression molding. The HIDP encapsulated by very-high-bond (VHB) tape can be used as display in harsh environments, such as low temperature (− 7.5 °C), high temperature (90 °C) and underwater, as shown in Fig. S5. In addition, the similar mechanical properties of the SFIE and the light-emitting layer, and good interface bonding of these two materials enable the HIDP to maintain a stable light emission during the stretching process, as shown in Fig. 2a and Movie S1. Figures 2a and S6 show that when the tensile strain is lower than 400%, the brightness of HIDP remains constant, showing a bright blue color. Only after the strain exceeds 500%, the luminous intensity of HIDP gradually weakens with the increase of strain. Even when the strain is as high as 700%, only 64% of the luminous intensity is lost, as shown in Fig. S6a.

**Fig. 3:**
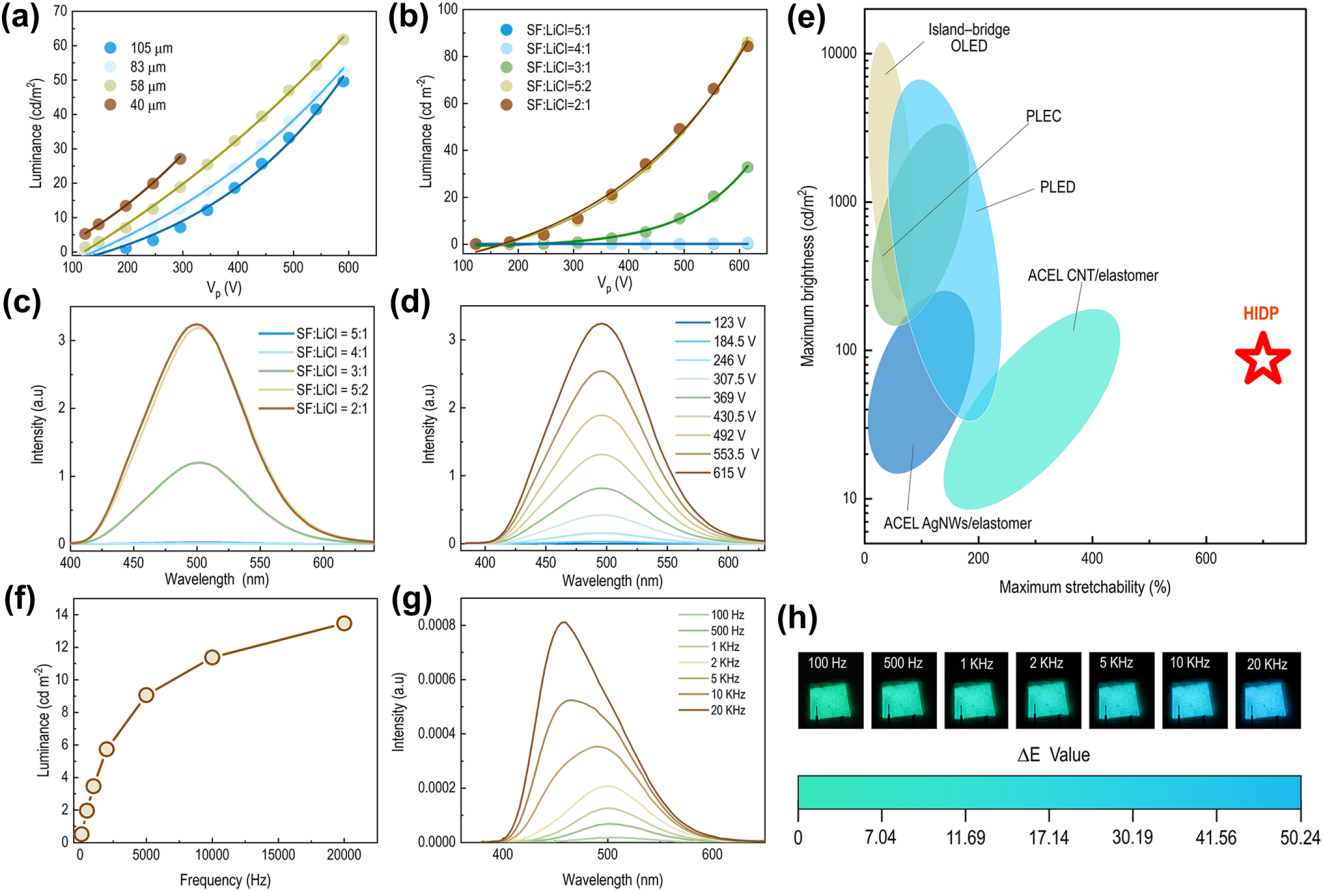
Luminescence properties of the HIDP. **a** Luminance of HIDPs versus excitation voltage with different thickness of EL film at various spin-coating rates. **b** Luminance of HIDPs versus excitation voltage at different mass ratios of SF/LiCl. **c** Emission spectra of HIDPs at different mass ratios of SF/LiCl when the excitation voltage is 615 V. **d** Emission spectra of HIDPs at various excitation voltages. **e** Comparison of the stretchability and maximum brightness of the HIDP, and other representative light-emitting devices. **f** Luminance-voltage frequency curve of the HIDP. **g** Emission spectra of the HIDP. **h** Snapshots showing the color change of the HIDP with the increase of the voltage frequency. The mass ratio of SF/LiCl in the HIDP is 5:2, and the excitation voltage is 200 V

The similar mechanical properties of HIDP and bioelastomers are further confirmed by tensile tests shown in Fig. 2. Its true stress–strain curve features a J-shaped increase in stress as the strain increases, i.e., $\frac{d\sigma }{d\varepsilon }>0$ (Fig. 2b). This curve shows that initially, a small increase in stress results in large extensions. However, the material becomes stiffer and more difficult to extend at larger extensions. J-shaped stress–strain curves cause the biomaterials to be extremely tough [48, 49]. This is because the lower part of the J-shaped curve is substantially extended for low applied stress. Thus, the shear modulus in this region is very low, and the released strain energy on the fracture cannot be transmitted to the fracture zone. The similarity between HIDP and bioelastomers is also reflected in the tensile toughness and Young's modulus, which are equal to 3.3 ± 1.3 MJ m^−3^ and 0.83 ± 0.48 MPa, respectively (Fig. S7). These values are equivalent to the skins of humans [50], pigs [51] and rats [52], and also match the requirements for e-skins. In addition, the elongation at break, 849 ± 80%, is even higher than that of bioelastomers and other soft materials, as shown in Fig. 2c. The maximum tensile strain at which the luminous intensity of the device can remain stable (defined as the effective working strain here) is 410% for HIDP. The HIDP is also 1.1–12.7 times higher than other flexible display devices, like PLEC [18, 22, 53], PLED [54–57], island bridge OLED [17, 58, 59] and ACEL AgNWs/Elastomer [19, 21, 27, 60, 61], as shown in Fig. 3e. The effective working strain of HIDP is even higher than the fracture strain of most bioelastomers, as shown in Fig. 2c. This advantage ensures the application of HIDP in areas that require large deformation, such as wearable sports devices, and saving of space during storage and transportation.

The HIDP also exhibits outstanding elasticity. When it is stretched to three times the original length, it can recover 90% within 10 s and 100% within 1 min, as shown in Fig. S4b. As shown in Fig. 2d, the HIDP maintains a stable light emission after 100 stretch cycles at the strain of 200%, as shown by Movie S2. In addition, no changes in luminescence intensity are observed as shown in Fig. S6b, indicating that the HIDP can withstand cyclic stretches. During a single stretch cycle, the ratio of dissipated energy, i.e., the area between loading and unloading curves to the work applied, i.e., the area under loading curves of HIDP is 0.44, which is a hysteresis value consistent with previously reported high-performance double network (DN) elastomers [62] and polyacrylamide (PAM) alginate hydrogels [63], as shown in Fig. 2e.

Mechanical fatigue and local damage may occur in the long-term use of luminescent display devices. Therefore, the fracture mechanics of HIDP are also evaluated by the cyclic stretch of single-edge pre-notched splines (Figs. 2f, g and S8). As Fig. 2g shows, the stress–strain curve of HIDP exhibits an n-shaped ductile fracture mode, which is essentially different from the linear response of brittle materials. Nevertheless, the fracture strain is still as high as 400%, even in the presence of a pre-notch. The pre-notched HIDP maintains a stable luminous state throughout the tensile fracture process, as shown in Figs. 2f, g. Its fracture toughness of 863 J m^−2^ is higher than that of pure PDMS elastomers with bimodal networks [64] (65 J m^−2^) and comparable to those of DN elastomers [62] (2,000 J m^−2^), and DN hydrogels [65] (2,113 J m^−2^) reported previously (Fig. 2e). In addition, it maintains a stable luminescence state during the 40 cycles of stretch with a maximum strain of 200%, as shown in Fig. S8. The fatigue threshold of the HIDP calculated by the cyclic stretching of pre-notched splines is 358 J m^−2^, as shown in Fig. S9. These values are comparable with DN PAAm hydrogels [66] and polyamide (PA) hydrogels [67], as shown in Fig. S10.

Furthermore, the cyclic stress–strain curve reveals that the pre-notched HIDP can maintain mechanical stability in long-term cyclic stretching, and the notch does not expand within this process, as shown in Fig. S8. In contrast, other ionotronics often suffer mechanical shakedown during cyclic stretching; materials experience decrease in structural instability-induced stress during the prolonged loading–unloading stretching. This mechanical failure often causes structural and functional instability of the devices.

In addition, there is a self-healable hydrogen bond between silk fibroin and water molecules in SFIE. The chelation between silk fibroin and lithium ions can also destroy and reformate. Accordingly, HIDP is self-healable. For example, after the HIDP is cut and then lapped, the HIDP can still emit with the same brightness as before cutting, as shown in Fig. S11. As illustrated in Fig. 2h, blue- and green-emitting HIDPs can be assembled into dual-color light-emitting devices taking advantage of this self-healing property. The devices maintain a stable two-color display under a high tensile stain, i.e., 300% (Fig. S12).

### Luminescence Properties of HIDP

As Figs. 3a, b show, the brightness $L$ of HIDP increases with the thickness of the emitting layer. When the thickness of the light-emitting layer is lower than 40 μm, the light-emitting layer will be damaged by the high electric field, as shown in Fig. 3a. In addition, the brightness of HIDP is also positively correlated with the applied voltage $V$, and this correlation can be quantitatively described by the impact ionization model. Specifically, according to the luminescence mechanism of HIDP, the charge at the interface enters the conduction band of the phosphor layer, and accelerates to high energy electrons under a high electric field. Susbequently, an inelastic collision takes place between the high energy electrons and the luminescent center. The electrons of the light-emitting center transition from the excited state to the ground state, and emits photons in this process [3, 23, 68]. Therefore, the voltage applied on the light-emitting layer is closely related to the brightness of the device. As Fig. 1a shows, the equivalent circuit of HIDP can be simplified as a structure of three capacitors in series. This is because when an alternating current is applied to both ends of the HIDP, it becomes equivalent to three capacitors in series. Among them, the electric double layer is formed by the copper electrode, SFIE contains the capacitors ${C}_{1}$ and ${C}_{3}$, and the light-emitting layer contains the capacitor ${C}_{2}$. As the capacitors are connected in series, they store the same amount of charge [69], i.e., $Q={C}_{1}{V}_{1}={C}_{2}{V}_{2}={C}_{3}{V}_{3}$. The capacitance tests reveal that ${C}_{2}$ is considerably smaller than ${C}_{1}$ in the range of 100 Hz-50 kHz, as shown in Fig. S13. Therefore, most of the voltage is coupled to the light-emitting layer. In this case, the relationship between $L$ and $V$ of HIDP can be quantitatively described as [70]:

2 $$L = L_{0} \exp \left( {\frac{ - \beta }{{V^{\frac{1}{2}} }}} \right)$$

where $L$ is proportional to $V$, and ${L}_{0}$ and $\beta$ are the physical constants of the light-emitting layer, which are related to the proportion and size of ZnS particles, the dielectric constant of the polymer matrix, and the device's thickness. As Fig. 3a, b shows, the quantitative description developed by this equation agrees closely with the experimental test values.

In addition to the luminescent properties of the light-emitting layer itself, the lithium content in SFIE also significantly affects the brightness of HIDP. As Figs. 3c and 3d show, when the mass ratio of LiCl in SFI increases from 5:1 to 2:1, the conductivity of SFIE increases from 2.1 × 10^–3^ to 43 × 10^–3^ S m^−1^ (Fig. S14), which increases the brightness of HIDP from 0.6 cd m^−2^ to 86 cd m^−2^ at a voltage of 615 V. In addition, as Fig. 3f shows, when the applied voltage is constant, the luminous brightness of HIDP increases as the voltage frequency increases. The CIE chromatogram and EL spectrum reveal that the wavelengths of the luminous peak of HIDP gradually shift from green (504 nm) to blue (460 nm) as the frequency increases from 100 Hz to 20 kHz. This can be noticed in Fig. 3g, h. The color change caused by the change in frequency is mainly attributed to the special energy level structure of ZnS:Cu. It has two energy levels of blue and green luminescent centers. At low frequencies, the holes captured by the green luminous center are dominant, which excites the green light. On the other hand, at high frequency, the combination of blue emission centers and holes increases, resulting in the blue shift of color.

### HIDP for Intelligent Identification and Question-and-answer Display

#### Audiovisual Interaction

**Fig. 4:**
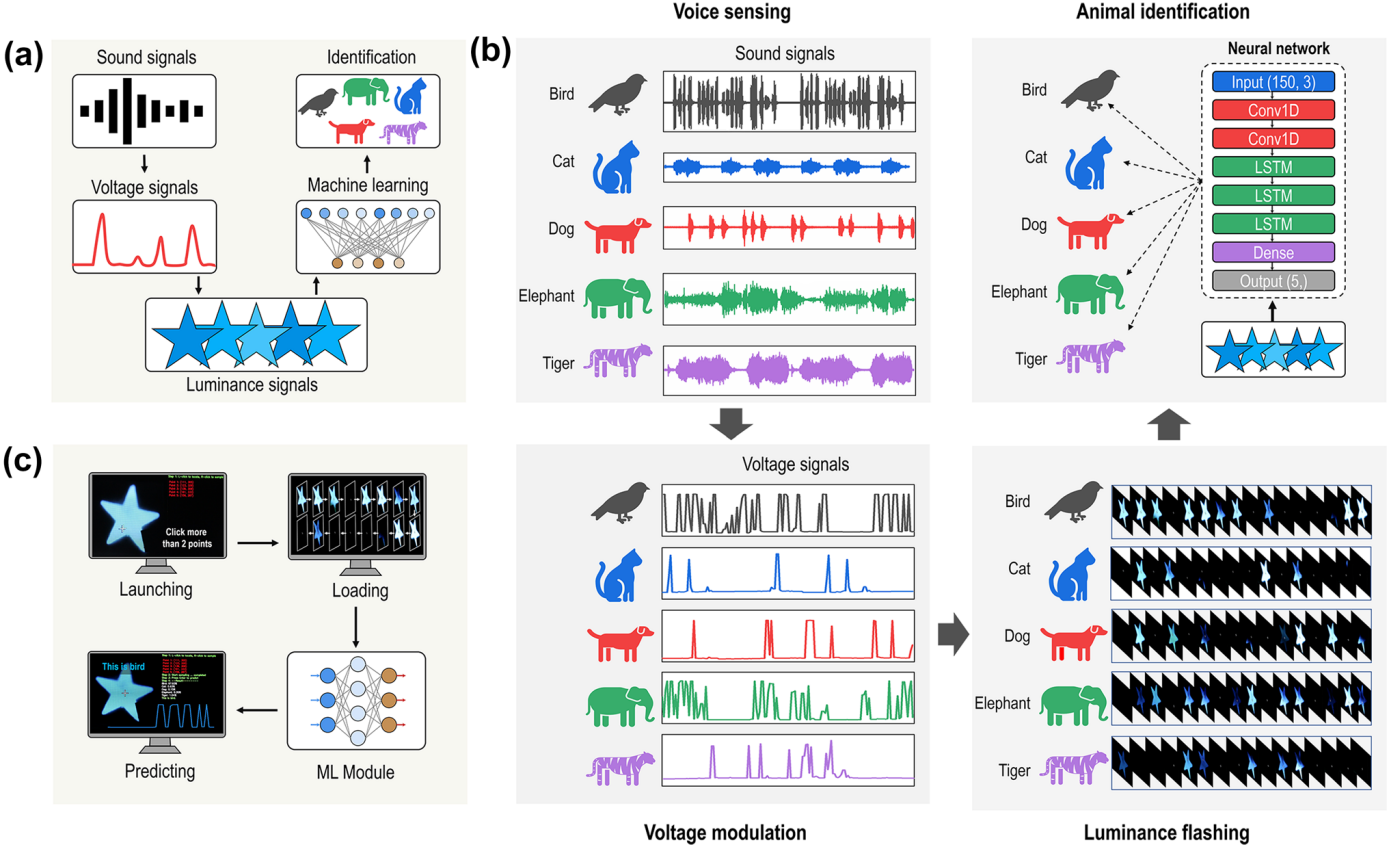
Visualization of sounds and intelligent identification via the HIDP. **a** Schematic of the technical roadmap for identification of animal sounds through monitoring and analysis of the luminescent change of the HIDP with machine learning. **b** Flow chart of sound visualization and intelligent identification of animal sounds through the HIDP. The sound signals are first converted into voltage signals to trigger the flash of star-patterned HIDP, which are recorded by a camera. Finally, the recurrent neural network is applied to identify the animal sounds from the RGB changes of interest points in flash patterns. **c** Schematic of operation steps for using sound visualization platform to distinguish the animal sounds. The operation steps include launching the movie, selecting five points in the HIDP area, loading of all the patterns in the flashing process, inputting the patterns into the ML model, and predicting the species of animal sounds

In real life, sound waves travel through a medium in a non-visual manner. Therefore, establishment of the correlation between sight and hearing is conducive to the interaction of the man–machine interface in a noisy environment and provides the possibility of communication for the audiovisually impaired individuals. This audiovisual correspondence can be established for HIDP because the brightness of the luminescence is proportional to the intensity and frequency of the voltage signal, as demonstrated in Fig. 4a. Therefore, different sound signals, e.g., volume and scale can be encoded into different voltage intensities and frequencies to drive the dynamic luminescence of the HIDP. Consequently, the HIDP can display brightness, color, flicker frequency, and other information corresponding to the sound to visualize it. Under this guidance, we realize the interaction between sound and display using the sound sensor and display driving chip. In addition, machine learning is used to learn the dynamic display process of different sound sources to convert the light to sound. The dynamic display of HIDP is used to recognize various sound sources.

The present study uses animal calls as the sound source for identification. As Fig. 4b shows, the sounds of different animals can be distinguished by the human ear because their sounds' frequency, amplitude, and timbre are different for different species and similar for the same species. Consider the calls of birds, dogs and cats as examples. The calls of birds are often short and sharp; thereby, the amplitude of these sounds mainly consists of sharp peaks, and the distance between the peaks is short. On the other hand, dog barking has a long and low tone, therefore, the sound signal has a constant wobble and there is a relatively large time difference between the two sounds. In addition, although the pattern of a cat meowing is similar to that of the dog barking in terms of amplitude changes, there are significant differences in signal strength and periodicity in the sound spectrum. When these spectra are converted into an applied voltage, it is found that their regularity is preserved even though the complexity of the spectra is greatly reduced. Therefore, the HIDP shows different periodic brightness changes for calls from different types of animals, while the calls from the same type of animal exhibit a significant similarity.

Accordingly, these brightness changes displayed by the HIDP are used as the input of machine learning classification. Using the recurrent neural network (RNN) model, we successfully identify the animal species based on the brightness variation information displayed by the HIDP. Sounds from five species of animals are included in this RNN dataset: birds, cats, dogs, elephants and tigers. Our compiled program can automatically capture 150 frames (30 frames per second) of luminance changes at points of interest in the HIDP display video stream corresponding to these animal calls. Features are pre-extracted through two one-dimensional convolution layers, and subsequently the classification results are obtained using three long short-term memory (LSTM) layers. The RNN synthesizes the required hidden information from the input changes and converges to about 100% accuracy after about 500 rounds of training. The classification model is further compiled into a graphical program to provide a user-friendly interactive experience, which allows the camera to either collect the HIDP brightness change video on the spot or locally retrieve the pre-recorded video from the PC. By manually or automatically selecting five points of interest in a video stream, the sound source can be identified from the brightness changes with an accuracy of about 100% for 200 rounds of random testing, as shown in Fig. 4c and Movie S3.

#### Voice-responsive Display

**Fig. 5:**
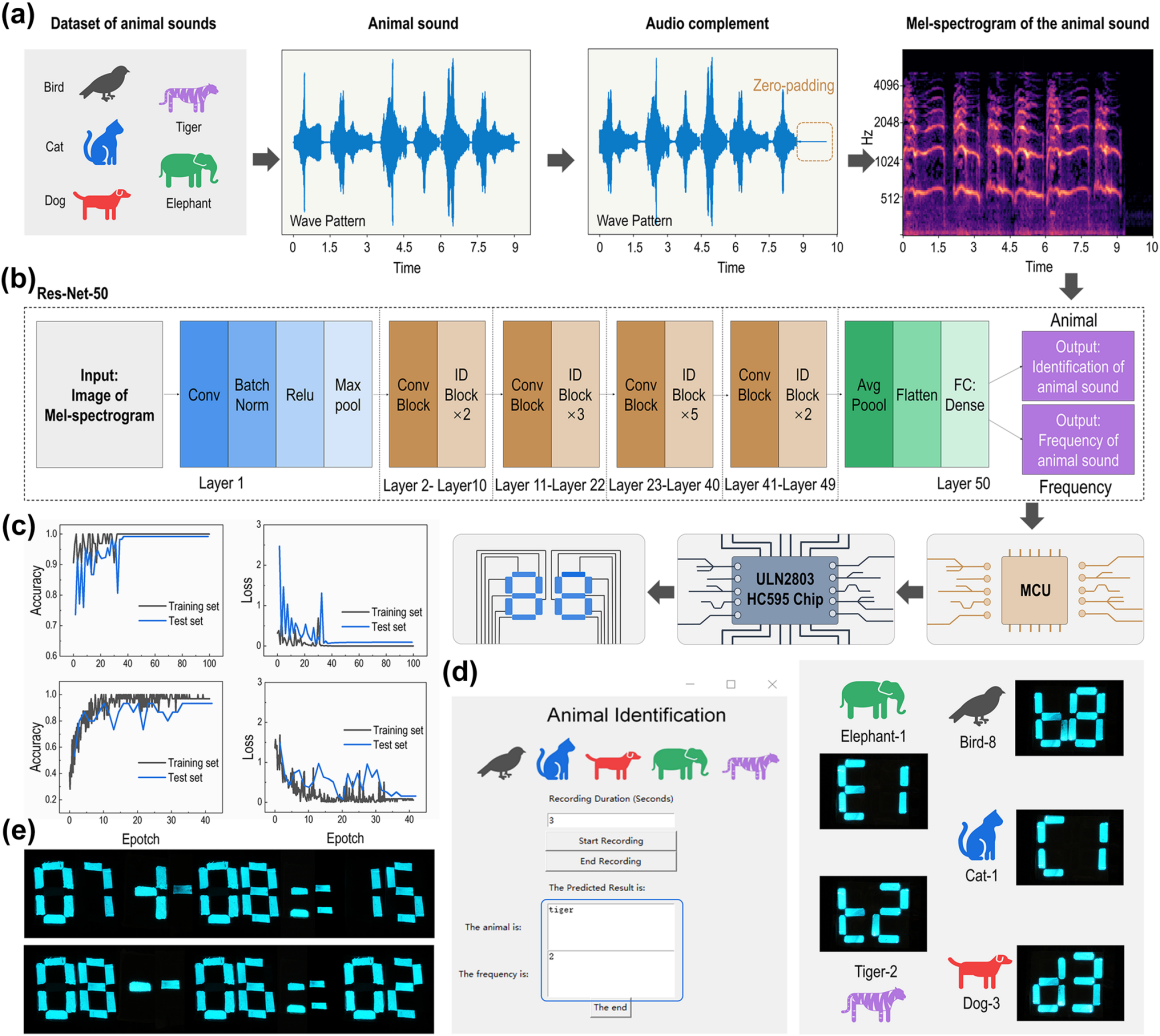
Identification of species and frequency of animal sounds through sound analysis which is accomplished using CNN models. **a** A flow chart of audio signal processing for establishing the dataset for the CNN models. **b** Structure of the CNN classification models based on Res-Net-50. **c** Accuracy and loss of the CNN models. **d** Tkinter interface and digital display of the HIDP showing that the sound identification platform can identify the species and frequency of animal sounds in real-time. **e** Snapshots exemplifying that the digital display of the HIDP can recognize the human voice and display the audiovisual interaction information through an intelligent question-and-answer approach

In addition, the species and frequency of animal calls can also be identified by learning the characteristics of the sound. In this case, a convolutional neural network (CNN) model was utilized, which takes the Mel spectrogram of animal sounds as input. The Mel spectrogram logarithmically renders frequencies above a certain threshold, which is known as the corner frequency. For example, in the linearly scaled spectrogram, the vertical space between 1,000 and 2,000 Hz is half of the vertical space between 2,000 and 4,000 Hz. However, in the Mel spectrogram, the space between those ranges is approximately the same. This scaling is analogous to human hearing; thus, it is better suited for applications that need to model human hearing perception and are used in audio classification. Here, 4586 Mel spectra of animal calls are used to train the CNN model and further predict the species and frequency of animal sounds, respectively (Fig. 5a). The constructed CNN model is based on the Res-Nets-50v2 [71], which is a 50-layer CNN that contains 1 MaxPool layer, 48 convolutional layers, and 1 average pooling layer. The Adam optimizer is used with a learning rate of 0.001 (Fig. 5b). In each epoch, the CNN model is fed by the Mel spectrogram having a format of 128 × 627. After tens of thousands of epochs, the identification accuracy of the models for species and frequency of animal calls converges to 99% and 93%, respectively, as shown in Fig. 5c.

To realize human–computer interaction, the CNN models are integrated into the tkinter-sourced human–computer interface illustrated in Fig. 5d. In this platform, the audio signal of animal sounds collected by the microphone is first transmitted to the computer terminal and automatically converted into a Mel spectrogram by employing the librosa library in Python [72]. The Mel spectrogram is further input into the CNN model for recognition. The recognition result, i.e., the species and frequency of animal calls, is displayed in real-time on the tkinter interface. Simultaneously, the single-chip microcomputer connected to the PC converts the recognition result into "utf-8" format to drive the HIDP and display the recognition result in real-time. As Fig. 5d shows, when the microphone receives two roars of a tiger, the model can recognize the signal and display "t 2" in HIDP and "tiger 2" on the tkinter interface in real-time (Fig. 5d, Movie S4). In addition to animal sounds, this platform can distinguish simple human sentences and analyze the semantics, enabling the HIDP to realize a question-and-answer display. As shown in Fig. 5e and movie S5, when speaking “7 plus 8 results” into the microphone, the HIDP not only displays “7”, “ + ”, “8”, but also displays the result of “15” in real-time. Similarly, when speaking “8 minus 6 results” into the microphone, the HIDP displays “8”, “–”, “6”, and the result of “2” sequentially.

## Conclusions

In summary, this study developed an electroluminescent HIDP with structural and mechanical properties that were highly skin-like. Structurally, the HIDP featured a sandwich structure comprising a middle light-emitting layer and two surface electrodes. In this design, the light emitting layer was constructed by embedding ZnS particles in silicone elastomer due to its high stretchablility and resilience. The two surface electrodes were made of SFIE, a material with composition, mechanical properties, and functions highly similar to those of human skins. Mechanically, HIDP, consistent with human skins, exhibited high stretchability, resilience, and self-healing ability. For example, the tensile strain of HIDP could reach 700% and recover to its original length within 1 min while maintaining a stable luminescence. In addition, after 100 cycles of stretching of a pre-notched HIDP with a maximum strain of 200%, no fracture occurred, and a constant luminous intensity was maintained. These mechanical merits and the inherent advantages of ionotronics in frost resistance and self-healing capacity allowed the HIDP to be used in extreme environments, such as low temperature, high temperature, and water, or complex mechanical loading, such as stretch, squeeze and crimp. More importantly, the advantages of HIDP were also reflected in its functionality and intelligence. The numerical correlation between the amplitude change of the animal sounds and the change in brightness of the display and the frequency of the brightness changes were established by combing with IoT and machine learning techniques. This allowed the HIDP to realize the audiovisual interaction and successful identification of the animal species from the brightness variation information displayed by the HIDP. The identification accuracy for sounds from five species of animals reached about 100% for 200 rounds of random testing. Additionally, by analyzing sound characteristics, the species and frequencies of animal sounds were identified, and HIDP displayed the recognition results in real-time. The accuracy for species and frequency identification reached 99% and 93%, respectively. Summarizing, this study provided a rational route for designing intelligent display devices for audiovisual interaction with the potential to accelerate the application of smart display devices in human–machine information interaction, soft robotics, wearable sound-vision system and medical devices for hearing-impaired patients.

### Supplementary Information

Below is the link to the electronic supplementary material.

Supplementary file1 (MP4 7562 kb)

Supplementary file2 (MP4 8825 kb)

Supplementary file3 (MP4 9006 kb)

Supplementary file4 (MP4 9163 kb)

Supplementary file5 (MP4 6195 kb)

Supplementary file6 (PDF 2168 kb)
