# Supplementary material for: Humanoid Intelligent Display Platform for Audiovisual Interaction and Sound Identification
Source: Nanomicro Lett. 2023 Oct 9;15:221. doi: 10.1007/s40820-023-01199-y (PMC10562358; doi:10.1007/s40820-023-01199-y)
Supplement: Supplementary file 6 — Supplementary file6 (PDF 2168 kb) [file 40820_2023_1199_MOESM6_ESM.pdf]

Supporting Information for

Humanoid Intelligent Display Platform for Audiovisual Interaction and Sound Identification

Yang Wang[1, †], Wenli Gao[1, †], Shuo Yang[1], Qiaolin Chen[2], Chao Ye[3], Hao Wang[1], Qiang Zhang[4], Jing Ren[1], Zhijun Ning[1], Xin Chen[2], Zhengzhong Shao[2], Jian Li[1, 5], Yifan Liu[1, 5], Shengjie Ling[1, 5, *]

[1]School of Physical Science and Technology, ShanghaiTech University, 393 Middle Huaxia Road, Shanghai, 201210, P. R. China

[2]State Key Laboratory of Molecular Engineering of Polymers, Department of Macromolecular Science, Laboratory of Advanced Materials, Fudan University, Shanghai, 200433, P. R. China

[3]School of Textile & Clothing, Yancheng Institute of Technology, Jiangsu 224051, P. R. China

[4]School of Textile Science and Engineering, Wuhan Textile University, Wuhan 430200, P. R. China

[5]Shanghai Clinical Research and Trial Center, 201210, Shanghai, P. R. China

[†] Yang Wang and Wenli Gao contributed equally to this work.

*Corresponding author. E-mail: lingshj@shanghaitech.edu.cn (Shengjie Ling)

Supplementary Figures

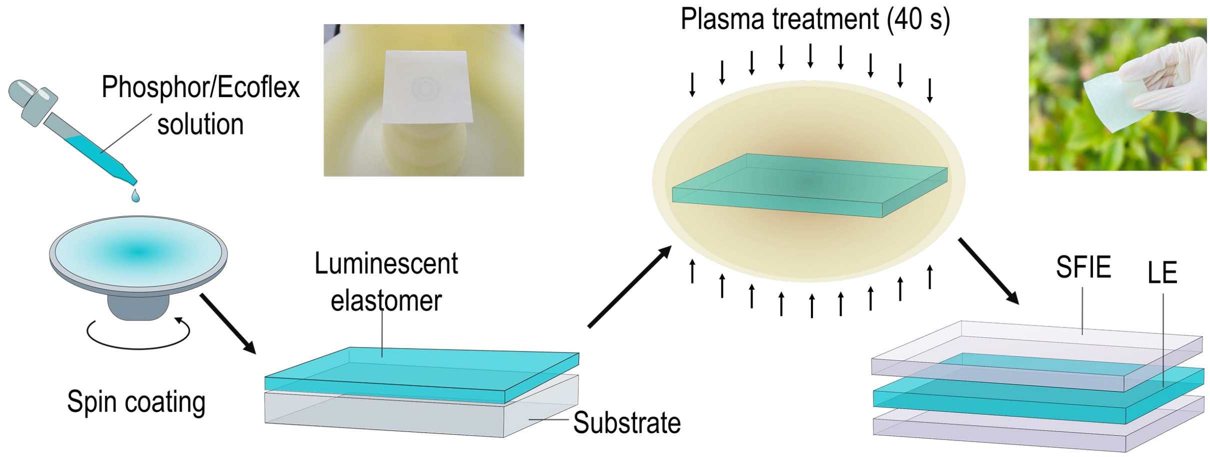

Fig. S1 Fabrication of HIDPs includes spin-coating of phosphor/ecoflex solution, plasma treatment of luminescent elastomer and assembling of SFIE with luminescent elastomer

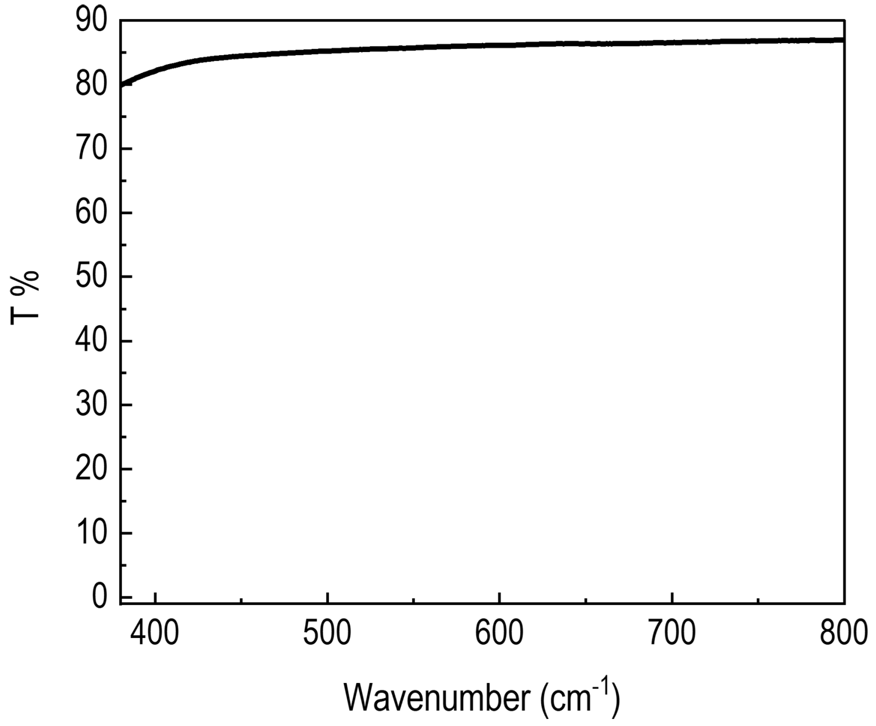

Fig. S2 The optical transmittance of SFIE when the mass ratio of SF/LiCl was 5:2

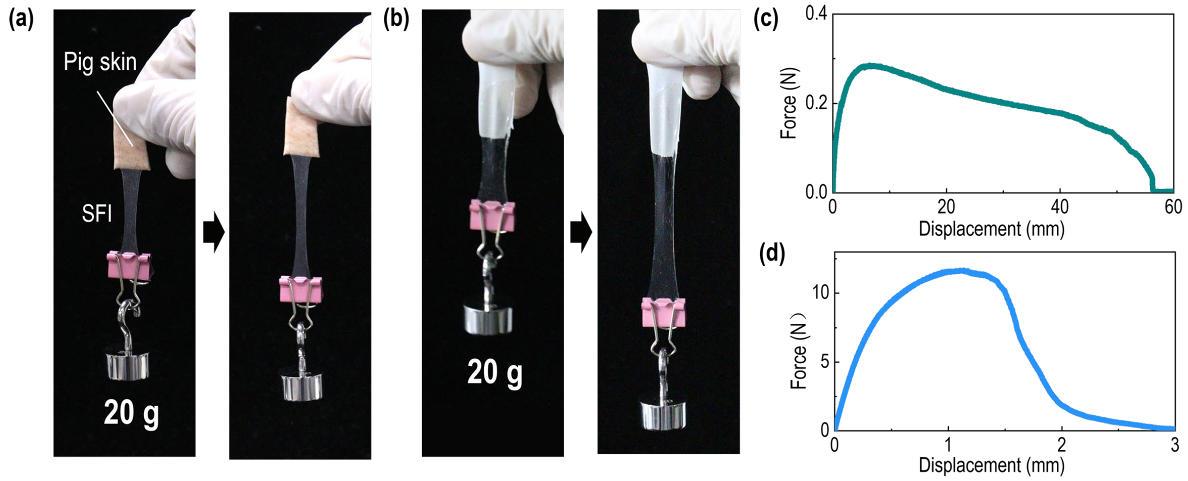

Fig. S3 a, b Photographs of adhered joint between SFIE and pig skin (**a**) or EL layer (**b**) that can hold a weight of 20g, which is 200 times its own weight. And the bonding interface can keep stable during the stretching process caused by the gravity of the weight. **c, d** Force-displacement curve of peeling test between pig skin and SFIE without (**c**) and with (**d**) rigid PS substrate

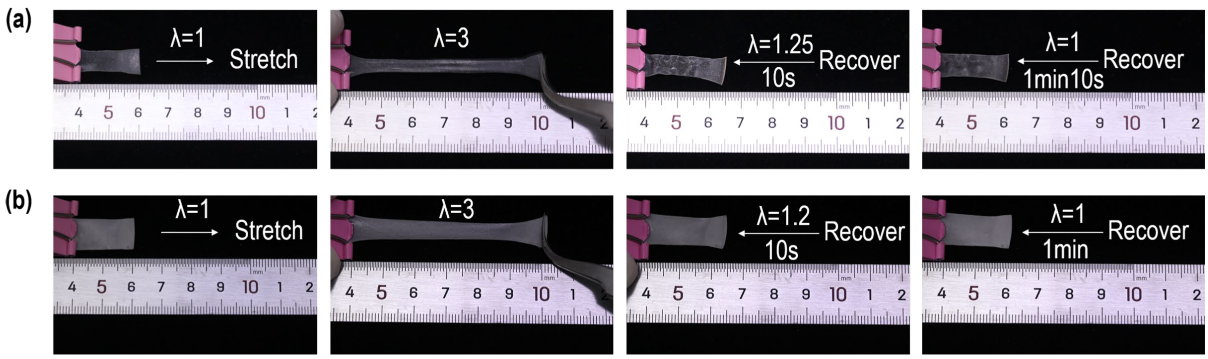

Fig. S4 a Photographs showing the elasticity of SFIE, which recovered 87.5% within 10 s and fully recovered after 70s. **b** Photographs showing the elasticity of HIDP, which recovered 90 % within 10 s and fully recovered after 60 s

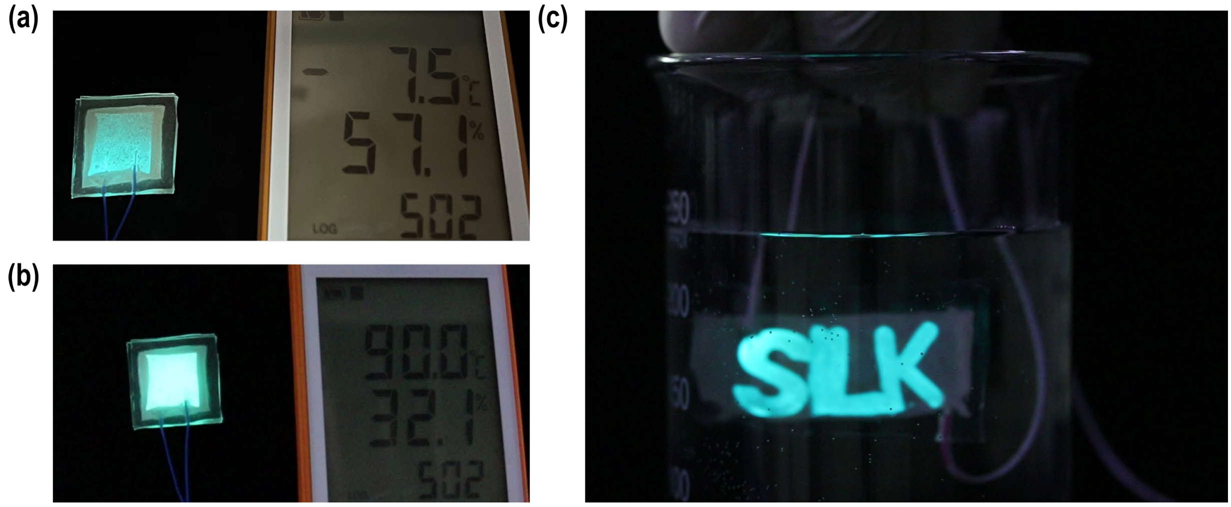

Fig. S5 Photographs showing HIDPs encapsulated by VHB glue can work at low temperature (-7.5 °C), high temperature (90 ° C) and underwater

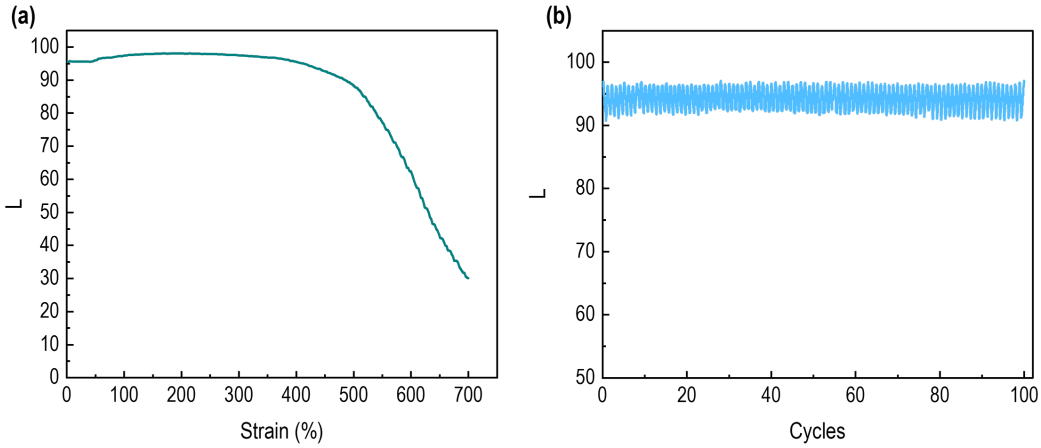

Fig. S6 a The brightness (L)-strain curve of HIDP during the stretching process with an excitation voltage kept at 492 V. **b** The brightness (L)-cycles curve of HIDP during 100 stretch cycles with stretch amplitude of 200% and an excitation voltage kept at 492 V

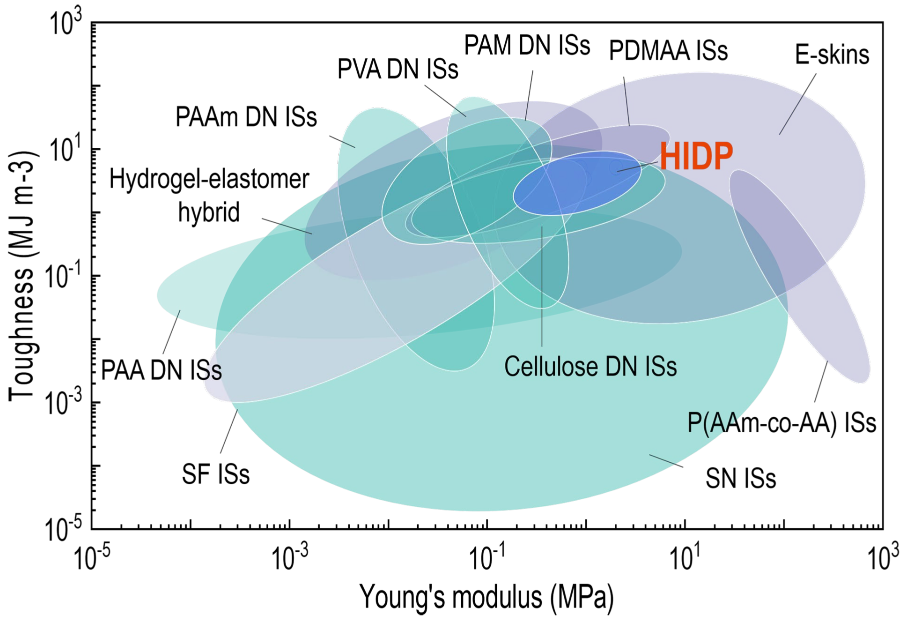

Fig. S7 Comparison of Young's modulus and toughness of the HIDP, and other representative ionotronics material. The Ashby plot of representative ionotronics materials has been adapted with permission [S1]. Copyright 2022, Royal Society of Chemistry

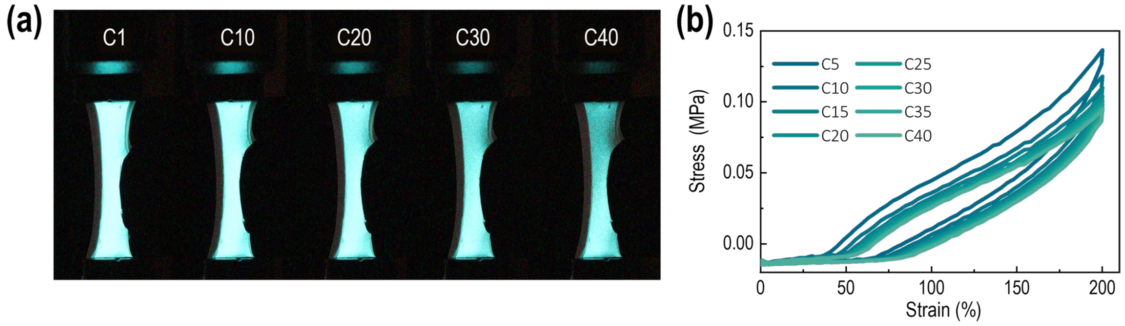

Fig. S8 a Snapshots of the pre-notched HIDP during 40 stretching cycles with a stretch amplitude of 200%. **b** Cyclic stress–strain curve of the pre-notched HIDP with a stretch amplitude of 200%

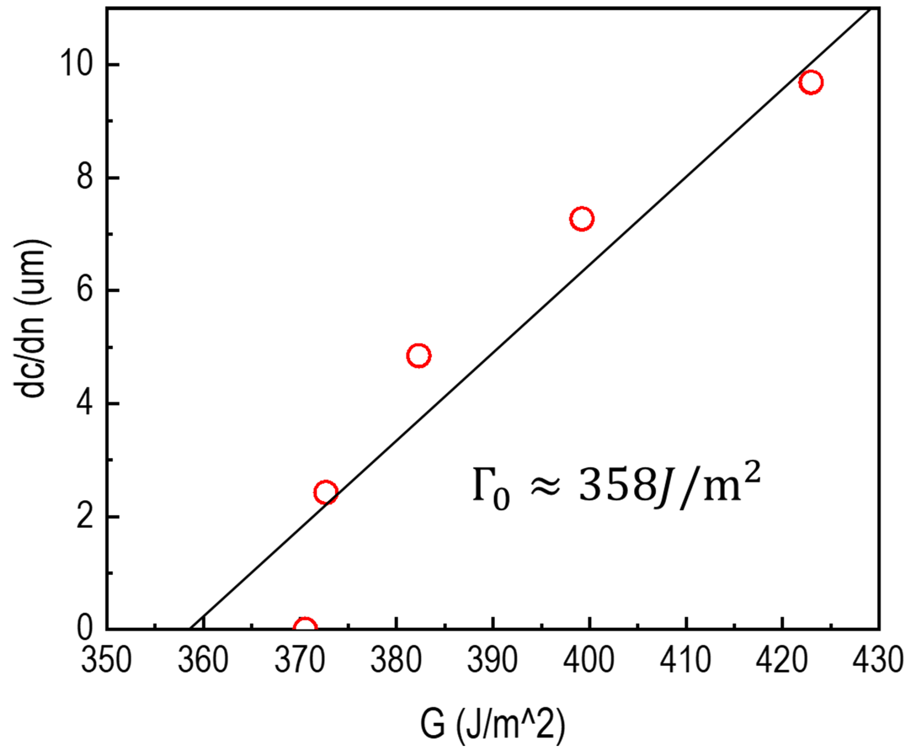

Fig. S9 Fatigue threshold Γ_0 of HIDP

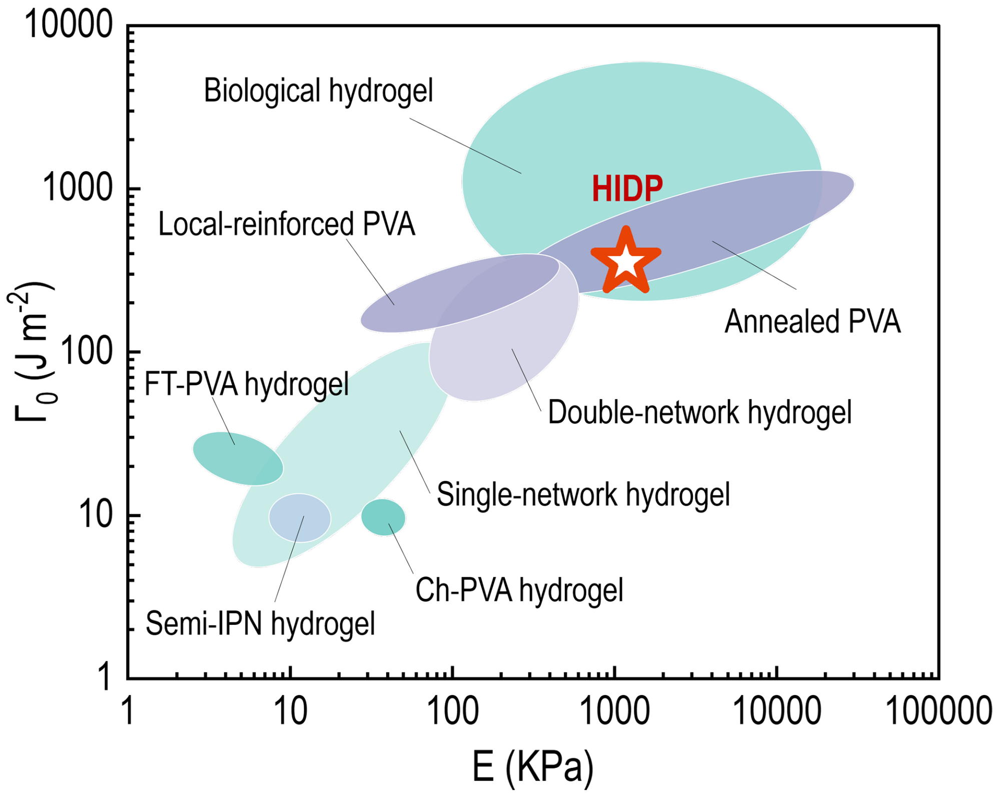

Fig. S10 Comparison of the Young's modulus E and □0 for SSIFs, and other representative soft materials. The Ashby plot of other elastomers has been adapted with permission [S2]. Copyright CC BY-NC 4.0

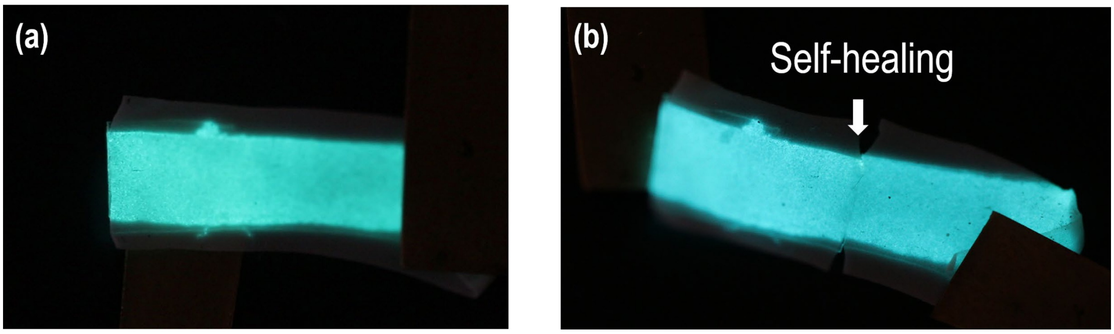

Fig. S11 Photographs showing self-healed HDIP can still emit with the same brightness (**a**) as before cutting (**b**)

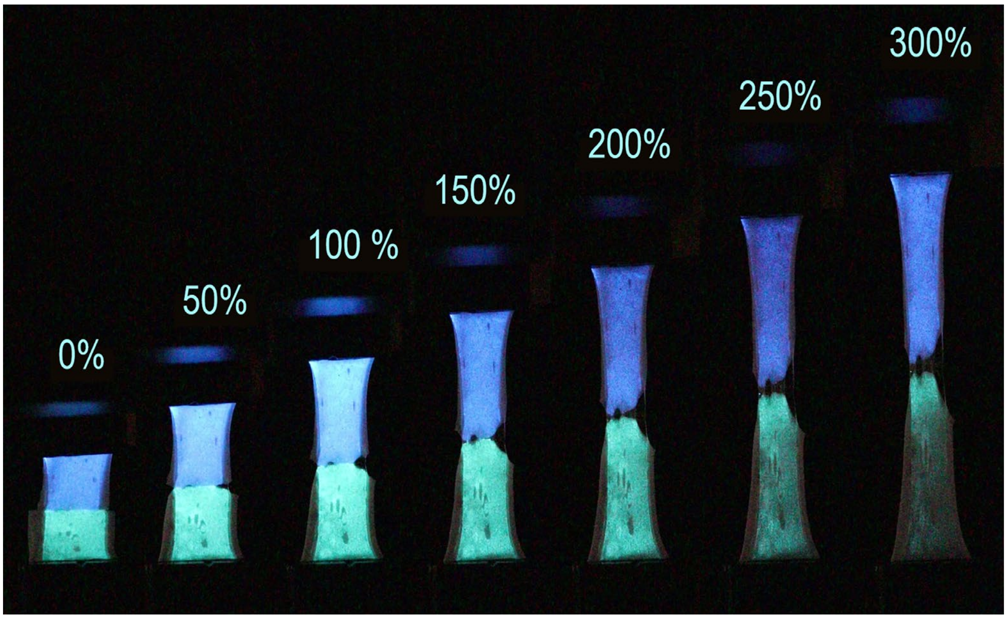

Fig. S12 Snapshots of the dual-color HIDP during the stretching process

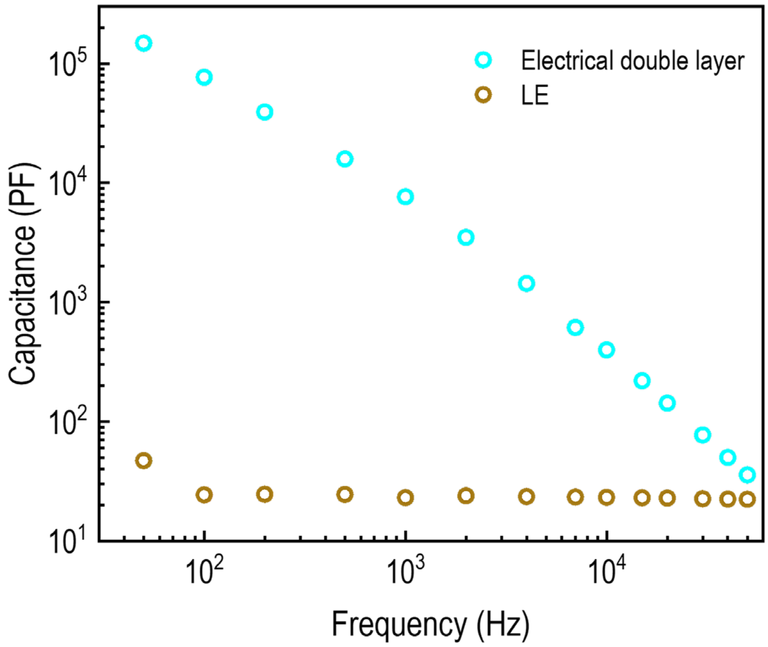

Fig. S13 The capacitance-frequency curve of electrical double layer formed by the copper electrode and SFIE, and luminescent elastomer (LE) layer

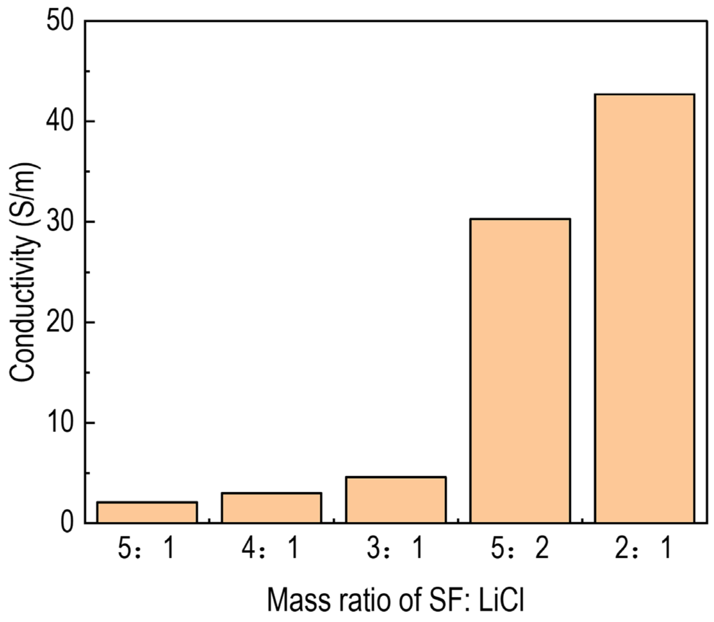

Fig. S14 The conductivity of SFIE versus mass ratio of SF: LiCl at a relative humidity of 44%

Supplementary References

[S1] C. Dai, Y. Wang, Y. Shan, C. Ye, Z. Lv et al., Cytoskeleton-inspired hydrogel ionotronics for tactile perception and electroluminescent display in complex mechanical environments. Mater. Horiz. **10**, 136-148 (2023). https://doi.org/10.1039/D2MH01034H

[S2] S. Lin, X. Liu, J. Liu, H. Yuk, H.-C. Loh et al., Anti-fatigue-fracture hydrogels. Sci. Adv. **5**, eaau8528 (2019). https://doi.org/10.1126/sciadv.aau8528
